# Innate Immune Signaling in Gliomas: Regulatory Mechanisms and Targeting Potential in Tumor Progression

**DOI:** 10.3390/life15101582

**Published:** 2025-10-10

**Authors:** Edmund Jung, Sara Al Jadidi, Christina Piperi

**Affiliations:** Department of Biological Chemistry, Medical School, National and Kapodistrian University of Athens, 11527 Athens, Greece; smg2300025@uoa.gr (E.J.); smg2200006@uoa.gr (S.A.J.)

**Keywords:** brain tumors, innate immune receptors, TME, toll-like, NOD-like, RIG-like, cGAS-STING receptors, scavenger receptors, C-type lectin receptors

## Abstract

Gliomas present as highly heterogeneous and aggressive central nervous system (CNS) tumors with challenging diagnosis and management. Traditional and current therapies are lacking efficacy in overcoming the complex and dynamic behavior of gliomas and the local tumor microenvironment. Emerging research highlights the significant role of innate immune receptors including Toll-like, NOD-like and RIG-like receptors, as well as cGAS-STING receptors, scavenger and C-type lectin receptors in glioma development and progression. These receptors can both impact immune modulation as well as facilitate tumor growth through interactions with tumor-associated macrophages, myeloid-derived suppressor cells and cytokine networks, contributing to immune evasion in the tumor microenvironment. Herein, we discuss the main signaling pathways induced through innate immune receptors in gliomas along with their functional properties in glioma pathology while exploring current applications to treatment. Utilizing innate immune receptors as therapeutic targets holds great promise, especially when used along with traditional chemotherapy and radiation schemes, strengthening immune responses. Future studies focusing on the deeper understanding of innate immune receptors signaling and complexity are highly required to enable novel immunoregulatory treatment schemes for gliomas.

## 1. Introduction

Gliomas are the most common primary malignant tumors among the diverse group of central nervous system (CNS) neoplasms [1]. Neuroimaging, MRI, and immunohistochemistry are the standard diagnostic procedures for gliomas, monitoring the increased proliferation, microvascular activity and necrosis which are the tumor’s hallmarks [2]. Primary as well as secondary gliomas both fulfil these criteria, rendering them indistinguishable by histology.

According to the 2021 WHO CNS5 classification, diffuse gliomas are mainly defined based on molecular diagnostics, while maintaining the traditional methods of histology and immunohistochemistry [3,4,5,6]. Diffuse gliomas are divided into adult and pediatric types. Adult-type diffuse gliomas include astrocytomas (IDH-mutant, grades 2–4), oligodendrogliomas (IDH-mutant and 1p/19q codeletion, grades 2–3), and glioblastomas (GBM, IDH-wildtype, grade 4). Low-grade pediatric-type diffuse gliomas include diffuse astrocytomas (MYB- or MYBL1-altered), angiocentric gliomas, polymorphous low-grade neuroepithelial tumors of the young and diffuse low-grade gliomas (MAPK pathway-altered). Pediatric-type high-grade gliomas include diffuse midline gliomas (H3 K27-altered), diffuse hemispheric gliomas (H3 G34-mutant) and diffuse pediatric-type high-grade gliomas (H3- and IDH-wildtype) [2,3,4,5,6].

Among all primary brain tumors, 16% are constituted by GBM at a male-to-female proportion of about 1.6 [7]. By impacting 3.19 individuals out of every 100,000, the incidence rates have been found to uptrend alongside age, peaking at a diagnostic age of 75–84 years. In children, this incidence rate is closer to 0.8 per 100,000, making GBM one of the most prevalent tumor types in this age group [7,8].

The current treatment strategies are ineffective at long-term management due to the tumors’ genetic complexity and adaptability. For patients with GBM, the modern therapeutic approach includes safe surgical resection, radiation, and chemotherapeutic agents combined with temozolomide (TMZ) [9]. Total surgical removal of these malignant neoplasms is rarely possible due to the highly invasive nature of the tumors that causes significant neurologic morbidity and mortality [10]. Oftentimes, the location of diffuse intrinsic pontine gliomas (DIPGs) prevents any safe surgical attempts. Utilizing radiotherapy alongside TMZ remains as the frontline treatment for those with this diagnosis; however, the 5-year survival rate is below 5% measured from time of diagnosis [10,11].

Due to the lack of effective chemotherapy, radiotherapy by itself remains as the only modern treatment regimen for DIPG. Given that under 10% of patients suffering from DIPG survive beyond two years post-diagnosis, the mortality rate is much more dismal [12]. Another challenge to consider while treating GBM lies in the diversity of tumor cells with regards to their phenotype and genotype, as well as function. Many attempts to correctly recognize commonalities among the cellular processes at the molecular level have been unproductive [2,13], as many innate receptors are expressed both on glioma cells as well as immune cells with varying outcomes.

In this review, we highlight the importance of innate immune receptor signaling in gliomas in an effort to identify novel molecular targets that will enable the development of innovative therapeutic strategies to improve diagnosis and survival rates. We have conducted a focused search of the literature in the PubMed, Scopus, and Web of Science databases, using different combinations of keywords such as “glioma” or “glioblastoma” with “innate immune receptors”, “innate immunity”, “TLR”, “NLR”, “cGAS-STING”, “RIG-I”, “CLR” and “scavenger receptor”. The majority of studies cited were published after 2010, with selected works from 2000 onward included for foundational context. We have excluded conference abstracts, editorials and non-peer-reviewed sources, only selecting articles based on their relevance to innate immune receptors, their signaling mechanisms and gliomas, giving priority to original experimental studies along with relevant preclinical models and high-impact reviews.

## 2. Main Functions of Innate Immune Receptors and Their Roles in Glioma Development

### 2.1. Toll-like Receptors (TLRs)

The initial response towards microbe infiltration involves the TLRs of the innate immune system, a family of pattern-recognition-receptors (PRRs) which defend against microbes [14]. TLRs can differentiate pathogen-associated molecular patterns (PAMPs) specific to microbial agents along with damage-associated molecular patterns (DAMPs) released through cell damage [15]. Within the CNS microenvironment, TLRs can be found within neuronal cells and glial cells, as well as various immune cells including T-cells, dendritic cells (DC), natural killer (NK) cells, monocytes, and macrophages [16,17].

They consist of ten receptors that are subdivided into surface TLRs (TLR1, 2, TLR4-6, and TLR10) which are found on the plasma membrane and bind to microbe-derived ligands along with intracellular TLRs, specifically 3, 7, 8 and 9 located in endosomes that detect genetic material originating via damaged cells and pathogens [14,18,19]. Intracellular localization of TLRs is crucial for preventing contact with self-nucleic acids, which could lead to autoimmune conditions [18].

**Figure 1:**
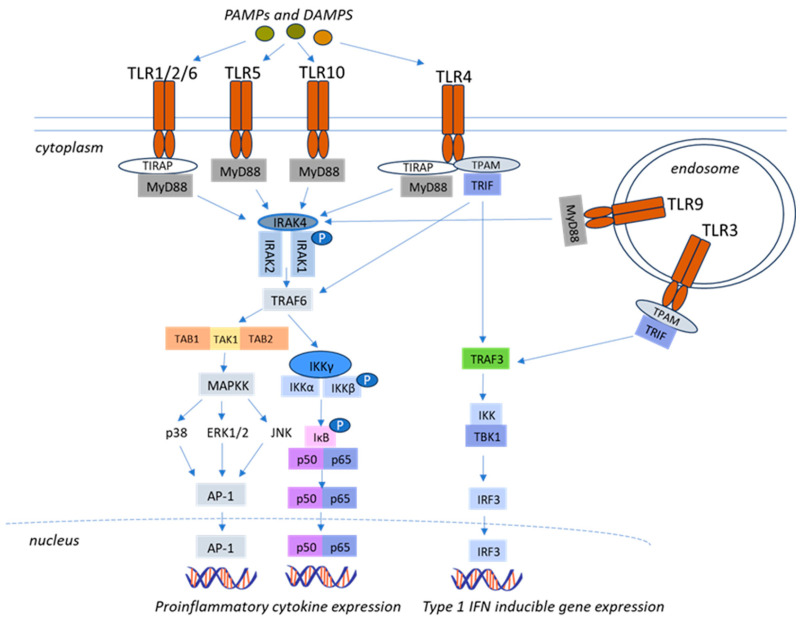
**Toll-like receptor (TLR) signaling pathways.** TLRs located on the plasma membrane detect pathogen-associated molecular patterns (PAMPs) and damage-associated molecular patterns (DAMPs) through adaptor myeloid differentiation factor 88 (MyD88). This leads to the activation of IL-1R-associated kinase-4 (IRAK4), and the subsequent phosphorylation of IRAK-1 and IRAK-2 [23], stimulates the tumor necrosis factor receptor-associated factor 6 (TRAF6). UBC13 and UEV1A, enzymes involved in ubiquitination, interact with TRAF6 to catalyze K63-linked polyubiquitination of TRAF6 itself and IkappaB kinase gamma (IKKγ, also known as NEMO or the nuclear factor-κB essential modulator) [24]. IKKγ, IKKα, and IKKβ form the IKK complex which phosphorylates IκB proteins, promoting their degradation and enabling NF-κB nuclear translocation [24,25]. TRAF6-mediated ubiquitin scaffolds also contribute to the activation of transforming growth factor beta-activated kinase 1 (TAK1) through its binding to TAK1-binding proteins such as TAB1. TAK1 either phosphorylates IKKβ to trigger NF-κB signaling or alternatively activates the mitogen-activated kinases (MAPKs) cascade, promoting p38 phosphorylation, extracellular signal-regulated kinases 1 (Erk1), Erk2, and c-Jun kinases (JNKs), which in turn activate the transcription factor activator protein-1 (AP-1) [24,25,26]. In contrast, the Toll/IL-1R domain-containing adaptor-inducing IFN-β (TRIF) dependent pathway engages in antiviral immunity by generating type I interferons (IFNs) and is activated by TLR3 and TLR4 [27,28,29]. After ligand binding, TRAF3 is mobilized by TRIF, which triggers the enzymes TBK1 as well as IKKε. These kinases phosphorylate interferon regulatory factor 3 (IRF3) and IRF7, enabling nuclear translocation and activation of interferon-stimulated genes [30]. Through its association alongside the serine/threonine kinase (RIP), TLR3 and TLR4 drive a second pathway that recruits TRAF6 and initiates a late phase of NF-κB signaling, consequently activating additional pro-inflammatory factors [27]. TLR4 integrates both MyD88 and TRIF signaling, inducing a more comprehensive immune response that includes both inflammatory cytokines and antiviral agents [20,29]. TLRs located in endosomal membranes (TLR3 and TLR 9) signal through TRIF and subsequently via TRAF3, TBK1 and IKKε to phosphorylate IRF3 resulting in type I IFN inducible gene expression [18,19,20,21]. Adapted with modifications from https://doi.org/10.1186/s42269-019-0227-2 [16].

Upon ligand binding, TLRs initiate a cascade of intracellular signaling pathways that regulate cytokine production and the promotion of inflammation. These processes are primarily mediated by two adaptor-protein-dependent pathways, namely the myeloid-differentiation factor 88 (MyD88) dependent and MyD88-independent pathways [20]. The primary signaling pathway involves the use of MyD88 while the alternative pathway can proceed through a MyD88-independent mechanism, utilizing the adaptor-inducing interferon-β found in the adaptor Toll-interleukin receptor domain [20,21]. With the exception of TLR3, the pathway dependent on MyD88 is crucial for initiating pro-inflammatory cytokine production and is employed by all TLRs (Figure 1) [22].

In gliomas, the roles of TLRe multifaceted and context dependent, involving both tumor-promoting and anti-tumor effects [20]. Elevated expression of TLR1, TLR2, TLR4, TLR5, TLR6, and TLR9 has been detected in GBM, particularly within the mesenchymal subtype with significant impact in tumor progression [20,21]. Moreover, TLR2, TLR3, TLR4, TLR7, TLR8, and TLR9 have emerged as critical modulators of glioma biology [17]. They can regulate inflammatory signaling and glioma cell proliferation, as well as tumor-associated immune cell activation. Their activity has been also associated with both resistance and responsiveness to therapeutic interventions [17,21].

More specifically, TLR2 contributes to glioma progression by fostering an immunosuppressive tumor microenvironment (TME) [17]. Upon heterodimerization with TLR1 or TLR6, it recognizes a wide range of ligands which include microbial, viral, and tissue injury-associated signals [17,31,32]. TLR2 activation in microglia has been shown to suppress CIITA and induce loss of MHC II expression via MAPK/ERK1/2 signaling, contributing to immune evasion in gliomas [21]. Furthermore, TLR2 signaling promotes the recruitment of immune cells such as regulatory T-cells alongside myeloid-derived suppressor cells (MDSCs), facilitating tumor immune evasion by interfering with the innate immune response mechanisms [29].

TLR4 activation has been predominantly linked to glioma progression and invasion via cytokines, chemokines, and type I IFN production [33,34]. In GBM, the presence or absence of the tumor suppressor gene *PTEN* biases TLR4 usage and internalization as well as MyD88 signaling [17,35]. PTEN regulates the Toll-interleukin-1 receptor (TIR) domain-containing adaptor protein (TIRAP), an adaptor that recruits MyD88 to TLR2 and TLR4 at the plasma membrane. This further affects the way that TLR4 is internalized inside the cell [36,37]. Studies have shown that numerous high-grade IDH-wildtype gliomas have mutations or deletions in *PTEN*. In the absence of PTEN, there is lesser control over TIRAP, resulting in stronger signaling through the pro-inflammatory MyD88-dependent NF-κB pathway which can potentially promote tumor growth [38]. Further studies have shown that TLR4 signaling may suppress apoptosis through blocking of caspase-9 activation [35,39].

TLR7 can further modulate immune response and glioma cell growth. It is expressed in glioma cells as well as in other immune cells such as microglia and macrophages inducing the production of pro-inflammatory cytokines and chemokines. TLR7 signaling can also suppress anti-tumor immune cells through cytokine production, notably IL-10 [17,40]. However, some studies have indicated that agonists targeting TLR7 may activate innate immune pathways and boost anti-tumor responses, providing a potential avenue for therapeutic intervention [41]. The aggregation of regulatory T cells (Tregs), a subcategory of T cells, suppresses immune responses in gliomas through the activity of plasmacytoid dendritic cells (pDCs) [29]. pDcs show a poor response to TLR7/9 stimulation, which generally stimulates an immune response. As a result, T cell immunity is weakened and type I IFN release is insufficient [28,29].

Several studies indicate that TLR9 expression in gliomas has been associated with malignancy [42,43]. Glioma stem cells express high levels of TLR9, leading to STAT3 activation [43]. Moreover, TLR9-mediated tumor progression involves the implication of various chemokines, particularly CCL2 and CCL5, along with matrix metalloproteinases (MMP), such as MMP-2 and MMP-9 [44,45,46]. Furthermore, the insulin-like growth factor 1 (IGF-1) has been shown to trigger TLR9 expression in glioma cells through activation of hypoxia-induced factor 1 alpha signaling, which in turn stimulates IL-1β, IL-6, IL 8 as well as CXCR4 [17,44]. In neuroinflammation, TLR9 expression is also remarkably elevated. CpG DNA has been shown to strongly stimulate the production of IFN-α in DCs that overexpress TLR9 to further accelerate the progression of GBM. While several studies report effects of TLR3, TLR7 and TLR8 signaling in gliomas, their overall roles remain context dependent and incompletely defined while the functions of TLR5 and TLR10 are largely underexplored [41,44].

### 2.2. NOD-like Receptors (NLRs)

Another distinct group of PRRs are the NOD-like receptors (NLRs), which are cytosolic sensors mainly expressed within immune cells including macrophages, monocytes and dendritic cells. Consisting of two parts, the nucleotide-binding domain and leucine-rich repeats (LRRs), they are also found in certain non-immune cells including tumor and epithelial cells. NLRs are stimulated through PAMPs and DAMPs [47]. Initiation of immune signaling pathways, including NF-κB, MAPK, IFN responses and inflammasome activation, depends on PRR sensing of PAMPs and DAMPs. Cytokines and chemokines are produced as a result of these signaling pathways that are vital in eliminating infections as well as damaged cells. However, excessive inflammation and inflammatory disorders are linked to these pathways being overactivated [48].

NLRs are divided into four subfamilies based on their N-terminal effector domains. These include the NLRP subfamily (pyrin domain, NLRP1-NLRP14), the NLRC subfamily (caspase recruitment domain also known as CARD, NLRC1/NOD1, NLRC2/NOD2, NLRC3, NLRC4, NLRC5), the NLRA subfamily (acidic transactivation domain or Class II Major Histocompatibility Complex Transactivator also known as CIITA) and the NLRB subfamily (baculoviral inhibitory repeat-like domain, or NLR family apoptosis inhibitory protein also known as BIR/NAIP). NLRX1 is sometimes separately categorized because of its distinct mitochondrial localization and the N-terminus targeting sequence [47,49].

The multi-complex protein inflammasome is a result of NLRs oligomerization with ASC adaptor proteins and precursor caspase-1 [47]. Inflammasomes trigger the innate immune response by activating caspase-1 and cleaving executor gasdermin D (GSDMD), secreting IL-1β along with IL-18 in the process. As a result, this creates membrane holes and initiates cell death, also known as pyroptosis [47]. As a result, the major downstream pathways of NLRs include NF-κB, MAPK, IFN and inflammasome activation [50].

In gliomas, inflammasomes can trigger neuroiflammation while their dysregulation has been involved in glioma development and progression [51]. NLRP3 activation in gliomas occurs in microglia and infiltrating macrophages but it can also be stimulated in tumor cells themselves. Stimuli also include extracellular ATP, reactive oxygen species (ROS), potassium ion efflux, crystals and nucleic acids, all of which can promote NLRP3 inflammasome assembly [51,52]. Without a CARD domain, NLRP3 is unable to activate the migration of procaspase-1 unless ASC protein serves as the adaptor [49]. Multiple agonists have the ability to stimulate NLRP3, and are classified either as self or foreign. Recent findings regarding the NLRP3 inflammasome regulatory mechanism have revealed that NLRP3 can trigger the PTEN/AKT and epithelial mesenchymal transition signaling pathways, which in turn induce glioma development, cell death, and spread [51]. Additionally, the NLRP3 inflammasome can regulate the NF-κB p65 and IL-1β signaling to induce glioma cell invasion and proliferation [51,52]. Of interest, the upregulation of the NLRC4 in astrocytes has been associated with the induction of an inflammatory TME in malignant gliomas and worse patient survival, indicating its diagnostic potential [53].

### 2.3. RIG-I-like Receptors

Retinoic acid-inducible gene (RIG)-I-like receptors (RLRs) comprise another unique PRR class with cytosolic RNA helicases which can detect specific PAMPs, RNA molecules released from stressed and dying cells as well as viral RNAs. Apart from the retinoic acid–inducible gene I (RIG-I), the RLR family also includes the melanoma differentiation-associated protein 5 (MDA5) and laboratory of genetics and physiology 2 (LGP2). RIG-I and MDA5 are involved both in PAMP detection as well as in downstream RLR signaling [54]. RIG-I can recognize 5’ double-stranded RNA as well as certain single-stranded RNA. In contrast, MDA5 mainly detects long dsRNA. Upon activation, their CARD domains interact with the mitochondrial anti-viral signaling protein (MAVS) found on the mitochondrial membrane, promoting MAVS oligomerization. This is scaffolded by TRIM and TRAF e3 ligases, which in turn recruit the noncanonical IKKs, TBK1 and IKKε [55]. These factors activate TF proteins IRF3, IRF7, and NF-κB, all of which lead to the production of pro-inflammatory cytokines and type I IFNs in gliomas [54,55,56]. The type I IFN signaling proteins trigger the interferon-stimulated genes (ISGs) through the activation of the Janus kinase signal transducer and activator of the transcription (JAK/STAT) signaling pathway. This cascade induces cancer cell apoptosis via an IFN-dependent or -independent mechanism [55,57]. Apart from their involvement in tumor cell death and stimulation of the innate immune system to generate interferons and pro-inflammatory cytokines, RLRs can also enhance the recruitment and activity of innate and adaptive immune cells (such as cytotoxic T cells), helping to overcome the immune-suppressive environment of gliomas. There is evidence that RIG-I and MDA5-induced chemokine/cytokine production in the TME activates several innate immune cells, such as NK cells and macrophages, while reducing regulatory T cell differentiation [55]. Altogether, RLRs can trigger tumor cell death but can also activate the anti-tumor immune response, suggesting their potential as therapeutic targets in glioblastoma [54].

### 2.4. STING Pathway (cGAS-STING)

**Figure 2:**
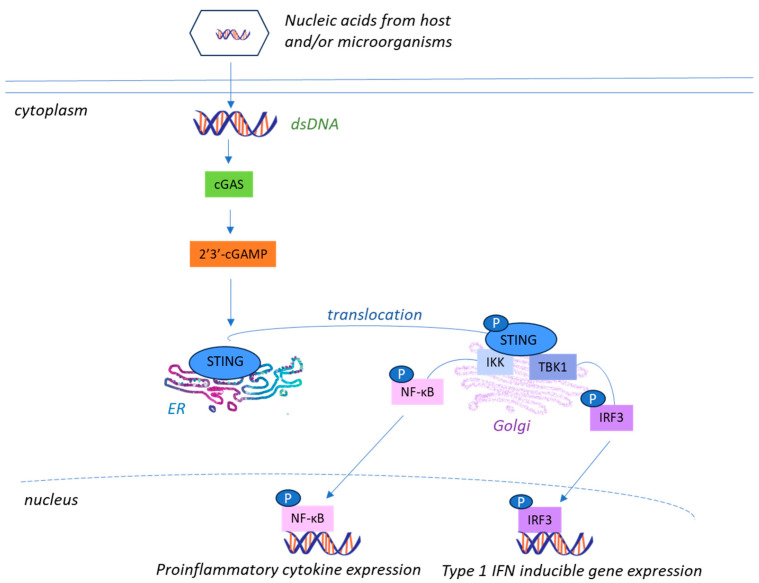
**The Cyclic GMP-AMP (cGAMP) synthase (cGAS) STING pathway.** Upon recognizing cytosolic DNA, cGAS generates 2′3′-cGAMP which initiates STING translocation to Golgi apparatus. It further interacts with TBK1-IFR3 signaling leading to the production of type I IFN inducible gene expression or IKK-NF-κB signaling to produce pro-inflammatory cytokine expression. Together, these pathways can subsequently trigger anti-tumor T cell responses [58,59,60] Adapted with modifications from https://doi.org/10.3390/jpm14070736 [61].

Like RLRs, the cGAS-STING, cyclic GMP-AMP synthase-STING pathway is also a key player in mediating innate immune system responses within gliomas. Cyclic GMP-AMP (cGAMP) synthase (cGAS) is categorized in the cytosolic DNA sensor subgroup of PRRs. Upon detection of cytosolic dsDNA, cGAS catalyzers the synthesis of 2′3′-cGAMP which binds to STING, an endoplasmic reticulum transmembrane protein (stimulator of interferon genes) and triggers its trafficking from the ER to the Golgi [58]. Following this activation, TANK-binding kinase 1 (TBK1) and IRF3 are phosphorylated, leading to type I IFNs and inflammatory agents, all of which contribute to the tumor immune response (Figure 2) [59,60].

Within the glioma TME, anti-tumor immunity is enhanced as STING activation drives the mobilization and action of dendritic cells, CD8+ T cells and macrophages. Type I IFNs produced via STING are critical in strengthening antigen presenting dendritic cells, ultimately stimulating CD8+ T cells [62,63]. In glioma patients, improved survival rates were associated with increased proliferation of CD8+ T cell (expression of Ki-67) and effector differentiation (marked by T-bet, the protein encoded by *TBX21* gene) [64]. Gliomas often suppress or downregulate molecules of the cGAS–STING pathway, diminishing IFN-I production and blocking immune surveillance. It was observed that pharmacological agonists that activate STING are correlated with a reduction in glioma progression [65]. Administration of cAIMP and CL656, a synthetic variant of cGAMP, was shown to improve T cell activation, reducing the progression of in-vitro gliomas in mice [66,67].

There is evidence that tumors have the potential to suppress both cGAS and STING expression [68,69] resulting in a hindered ability to detect DNA from the TME, which leads to a lowered immune response and ultimately continued growth of the tumor [70]. This suppression of STING arises via several mechanisms, including loss-of-function (LOF) in the cGAS and STING genes as well as epigenetic changes involving promoter DNA methylation [68,71]. A study found that eliminating cGAS or STING both resulted in suppressed immunity because of the absence of type I IFN [72,73].

While STING activation in the short term is anti-tumorigenic, continuous expression can have the opposite effect. A kinome array study showed that continuous STING activation can impact the vasculature of blood vessels in gliomas, resulting in fewer yet more dilated blood vessels, ultimately leading to induction of hypoxia and tumor growth. They also found that VEGF expression was increased in the tumors of experimental mice, highlighting the ambivalent nature of STING [65].

### 2.5. Scavenger Receptors

Scavenger receptors are PRRs abundantly expressed on myeloid cells such as macrophages, dendritic cells and microglia but are also found on endothelial, epithelial and other parenchymal cells. In some cases, they are intrinsically expressed by tumor cells. They recognize DAMPs by strongly binding to associated oxidative byproducts [74,75,76]. Advanced glycation end-products (AGEs) as well as the receptors for AGEs (RAGEs), are binding targets which activate pathways closely linked to the inflammatory response (Table 1) [77]. While scavenger receptors provide an innate protective mechanism, they can also contribute to a chronically inflammatory environment [76]. Current research has shown 12 different classes of scavenger receptors, namely SR-A to SR-L [75].

In the context of glioblastomas, SRs have been associated with glioma progression through their interaction with glial cells and the TME. SR-G, also referred to as CXCL16, facilitates the transmembrane uptake of OxLDLs (oxidized low-density lipoproteins) [78]. Compared to healthy brain tissue, researchers have detected higher levels of CXCL16 in GBM tissue with contributions from both glioma cells and tumor-associated microglia. The presence of CXCL16 derived from glioma cells was observed to promote tumor invasion and proliferation through a chemokine receptor that binds the soluble form of CXCL16, C-X-C chemokine receptor 6 (CXCR6) [78]. In contrast, CXCL16 was shown to exert anti-inflammatory effects in microglia. This finding highlights the context-dependent nature of the CXCL16-CXCR6 axis and how it can either be pro- or anti-tumorigenic depending on whether it recruits effector T cells or fuels lipid metabolism in tumor cells and TAMs [78,79]. TCGA data also showed improved survival in patients with CXCR6 deletions, along with poorer prognosis when this receptor is overexpressed, underscoring the importance of the CXCL/CXCR6 pathway in inflammation [78,79].

SR-J1, also known as RAGE, is an advanced glycation end product receptor and able to bind to numerous ligands [74,78]. A notable RAGE-binding DAMP is HMGB1, which is released during cell death [80]. RAGE and HMGB1 have been detected to colocalize on tumor-associated macrophages (TAMs). Blocking RAGE resulted in reduced inflammatory responses through the MAPK-ERK pathway [81]. TMZ treatment in human GBM facilitated increased HMGB1 expression, which was associated with better patient outcomes [78,82].

### 2.6. C-Type Lectin Receptors (CLRs)

C-type lectin receptors (CLRs) primarily detect carbohydrates found on pathogens such as fungi, viruses and various bacteria. Some CLRs, such as dectin-1, use spleen tyrosine kinase (SYK) to start a cascade of immune reactions. Other CLRs, such as DC-SIGN, use the RAF1 signaling pathway to antitumor response (Table 1) [83,84].

In gliomas, CLRs detect altered glycosylation patterns, DAMPs and fungal-like glycans and promote antigen uptake and modulate dendritic cell and microglial response.

However, CLRs are often exploited by tumor cells to evade immune responses. Gliomas can use CLRs to induce tolerogenic signals that suppress immune activation, thereby creating an immunosuppressive TME. For example, glioma cells can interact with CLRs on APCs, including macrophages or DCs driving tolerogenic signaling. This involves IL-10 production, SOCS upregulation, impaired antigen cross-presentation or metabolic reprogramming, all of which reduce the ability of APCs to effectively stimulate T cells (Table 1) [85].

**Table 1:** Distinct functions of innate receptors in glioma versus innate receptors in immune cells can exert varying roles depending on context.

Receptor	Tumor Cell Mechanisms	Immune Cell Mechanisms	References
TLR2	Drives tumor proliferation and invasion via NF-κB	Drives tumor progression via NF-κB, IL-6, TNF-α pathway, cytokine release	[17,29]
TLR3	Induces apoptosis via type I IFN but secondary pathway with TLR4 can bias toward pro-tumor		[27,28,29,30]
TLR4	Drives tumor proliferation and invasion via NF-κB, IL-6, TNF-α pathway, especially in absence of PTEN	Drives tumor progression via NF-κB, IL-6, TNF-α pathway, cytokine release	[17,35,36,37,38,39]
TLR7	Drives tumor progression via IL-10 but agonists shown to have anti-tumor potential		[17,28,40,41]
TLR9	Drives tumor proliferation and invasion via CCL2, CCL5 and MMP-1, MMP-9	Linked to various chemokines CCL2, CCL5 and MMP-1, MMP-9	[43,44,45]
NLR	Incudes pytoptosis via IL-1β, IL-18 from inflammasomes	Drives tumor progression as NLRP3 can trigger PTEN/AKT, NF-κB	[47,48,49,50,51,53]
RLR	RIG-I/MDA5 activation induces IFN production and apoptosis	Induces pathways which induce cytokine release and type I IFN expression	[54,55,56,57]
STING	Drives apoptosis through IFNs but can suppress both cGAS and STING expression	Drives apoptosis through type I IFNs, CD8+ T cell proliferation	[62,63,64,65,66,67,68,69,70,71,72,73]
Scavenger receptors	Binds AGEs and RAGEs to drive inflammation	Drives tumor progression when CXCL16-CXCR6 fuels lipid metabolism in tumor cells and TAMs but can induce apoptosis when CXCL16-CXCR6 recruits effector T cells	[74,75,76,77,78,79,80,81,82,86,87,88]
CLRs	Drives tumor progression as CLRs are used to include tolerogenic signals, creating an immunosuppressive TME	Dampen anti-tumor immunity via Tregs and MDSCs	[83,84,85]

Additionally, CLRs may contribute to glioma progression by promoting mobilization of immunosuppressive cell subsets, including Tregs and MDSCs, which further dampen antitumor immunity [89]. Such an interaction reduces the likelihood of an elevated cytotoxic T cell reaction, allowing the tumor to grow unchecked [89].

Emerging research suggests that targeting CLRs or their signaling pathways could represent a novel therapeutic approach to disrupt glioma-mediated immune evasion. Some types of CLRs may also serve as biomarkers of the TME, reflecting patterns of myeloid cell infiltration in gliomas. However, the dual role of CLRs in both immune activation and suppression underscores the need for careful modulation to avoid unintended effects on immune homeostasis [85].

Table 2 summarizes the main signaling pathways mediated by innate immune receptors in gliomas along with their pro- and anti-tumorigenic effects.

## 3. Cross-Talk of Innate Immune Receptors with Tumor Microenvironment

The detection of DAMPS and PAMPS by innate immune receptors drives a cytokine cascade. Through cytokine release, innate immune receptor signaling recruits tumor-associated macrophages (TAMs), enhances the expansion of MDSCs and indirectly shapes T cell responses.

### 3.1. Tumor-Associated Macrophages (TAMs)

TAMs comprise about 30–50% of glioma mass, mainly originating from resident microglia within the CNS and monocytes mobilized from peripheral circulation [103]. Macrophages are highly plastic, hence the immunosuppressive TME of gliomas can be remodeled by influencing TAM to raise the fraction of the pro-inflammatory subtype [103]. A distinction has been made between M2-type macrophages, which are abundant within the TME and exhibit pro-tumorigenic properties, and M1-type macrophages with anti-tumorigenic potential. This classification attempts to categorize TAMs based on phenotype and genotype [43]. Apart from cancer cell growth promotion, TAMs are also involved with inactivating T cells, potentially due to programmed death-ligand 1 (PD-L1) expression which results in further tumor immune evasion [103,104].

M1 and M2 macrophages are distinct with respect to the expression of receptors, production of inflammatory mediators, as well as signal transduction. IFN-γ and other microbial compounds drive mononuclear/phagocytic cells, which then differentiate into the M1 phenotype. Pattern recognition receptors on membrane-bound M1 macrophages, including TLRs, recognize microbial products and trigger the expression of receptors involved in antigen presentation, the development of pro-inflammatory cytokines such as TNF-α, IL-1B, and IL-12, upregulate antigen-presenting cell surface molecules, and improve autophagy [104]. IL-4, IL-10 and IL-13 activate mononuclear/phagocytic cells, which then develop into the M2 phenotype. Glioma-infiltrating myeloid cells including microglia (MG) and monocyte-derived macrophages (Mo-TAMs) can be polarized toward immunosuppressive states by tumor-secreted factors such as TGF-β and M-CSF, both of which can promote synthesis of anti-inflammatory mediators. Tumor-derived TGF-β microglia/microphages (MMs) can be polarized towards the M2 phenotype by tumor-derived factors such as TGF-β and M-CSF, which in turn can promote the synthesis of anti-inflammatory chemicals [105].

Studies have shown that M2 macrophages exert a dampening effect on immunity, through several mechanisms including the release of anti-inflammatory factors, mainly IL-10, IL-6 and TGF-β along with obstructing antigen presentation and associated molecules. MHC II, CD80 as well as CD86, all of which are related to cell surface presentation, show decreased expression, ultimately causing lymphocytes to undergo programmed cell death [106]. GBM cells secrete multiple factors that mobilize TAMs and drive differentiation towards the pro-tumorigenic M2-like phenotype as a result of secreted cytokines. Furthermore, TAMs are induced to release cytokines that further provoke tumor invasion and progression [107]. Glioma cells release the C-X-C motif chemokine ligand 16 (CXCL16), which differentiates TAMs to the M2 variant by signaling through CXCR6. Additionally, CXCL16 or CXCR6 signaling can directly support tumor progression and invasion [107]. GBM cells release glycoprotein osteoponton (OPN), which is also known to induce invasion, survival, and angiogenesis but at the same time stimulates IL-6 production in microglia, which affects glioma cells by enhancing their invasiveness [108]. Both TAMs and GBM cells are known to release TGF-β, another cytokine that promotes angiogenesis, invasion, and proliferation of cancer cells. Nonetheless, it is also recognized to contribute to the polarization process and the recruitment of TAMs to the TME [107].

After recruitment to the TME, TAMs secrete multiple signaling molecules and growth factors, all of which promote tumor growth. These mainly include the vascular endothelial growth factor (VEGF), angiopoietin 2 (ANG2), IL-6, along with IGF-binding protein 1 (IGFBP1), promoting angiogenesis [107]. Furthermore, it has recently been shown that TAMs invading glioblastomas with strong granulocyte infiltration express alternative proangiogenic factors. High levels of CD13 and CXCL2 expression in these TAMs may promote angiogenesis when VEGF is not present [105].

A study of a mouse glioblastoma model revealed that factors released by TAMs, notably CCL8, CCL5 and MMPs, contribute significantly to tumor invasion and growth through the breakdown of the extracellular matrix surrounding tissue [109,110]. GBM have chemokine receptors on their cell surface, including CCR1 and CCR5, while TAMs release CC chemokine CCL8. By binding to CCR1 or CCR5, CCL8 has been shown to enable formation of finger-like projections which increase tumor mobility and, ultimately, invasion. Furthermore, CCL8 triggers the pathways of ERK1 and ERK2, which increases the stemness of glioblastoma cells and causes invasion. Reduced invasion results from using ERK1 or ERK2 inhibitors as therapeutic agents [107,109]. CCL2 is found to be expressed in glioma, which causes nearby microglia to produce IL-6, resulting in a more infiltrative tumor [110]. The microglia then differentiate into M2-type macrophages, which support tumor growth through its suppressive effects and anti-inflammatory agents. Several studies indicate that the immunosuppressive M2-type subtype appears to dominate TAMs within malignant gliomas, often producing agents that inhibit immune responses, notably surface proteins and IL-10, TGF-β. Glioma cells produce angiogenic factors and tumor growth factors as well as remodeling of the ECM, which collectively enhance tumor growth [43,104].

It has also been demonstrated that glioma TAMs lack phagocytosis. The study of Rodrigues et al. showed that glioma cell-conditioned monocytes significantly reduced their capacity to phagocytose bacterial cell wall particles after stimulation [43,111]. Furthermore, they detected a decreased expression of cell surface molecules as well as the lack of T cell upregulation. Additional evidence demonstrated that human monocytes cause activated autologous T cells to undergo apoptosis after coculture with malignant glioma cells, which is a known consequence of insufficient communication between macrophages and T cells [43,111]. Immune checkpoint signaling, such as PD-L1, could also be a contributing factor to this effect [43].

There is strong evidence that STAT3, a key player in many immune inhibitory pathways, shows higher activation in glioma cells but also in TAMs, driving immune suppression. STAT3 is turned on by signals including IL-6, IL-10, epidermal growth factor and fibroblast growth factor, making it a key player in glioma immune evasion [43,112].

### 3.2. Myeloid-Derived Suppressor Cells (MDSCs)

MDSCs constitute key players of the glioma TME, suppressing antitumor immune responses and facilitating tumor progression. Gliomas secrete growth factors including VEGF and GM-CSF that recruit and expand MDSCs, although no single cytokine can fully account for their induction. Instead, a complex cytokine milieu appears to be needed as shown via blocking studies and the fact that M-MDSCs can be generated in vitro from monocytes with certain tumor line supernatants [113,114]. These cells inhibit cytotoxic T cell immunity via release of arginase-1, NO, and ROS, collectively impairing T cell function, and furthermore contributing to an immunosuppressive TME [113].

Additionally, MDSCs promote the production and release of anti-inflammatory cytokines, notably IL-10, which further dampens immune activity in the TME. One study revealed that MDSC levels are greatly elevated in the surrounding blood and tumor tissues found in glioma patients, showcasing a correlation with tumor progression and poor prognosis [115].

Innate immune receptor signaling is a key contributor to the modulation of MDSC activity in the context of gliomas. It has been shown that activation of TLRs, such as TLR4, in the glioma TME enhances MDSC recruitment and suppressive functions [113]. In parallel, when the NOD-like receptor protein 3 inflammasome (NLRP3) is activated, it stimulates pro-inflammatory cytokine production, mainly IL-1β, that indirectly enhances the expansion and activity of MDSCs [114]. MDSCs play a critical role in influencing glioma progression through hindering antitumor mechanisms and enhancing immunosuppressive aspects within the TME. Strategies aimed at targeting MDSCs or inhibiting their suppressive functions are currently being explored to enhance antitumor immunity in the context of gliomas [116].

### 3.3. Monocytes

Monocytes are considered as central mediators of glioma immunity, serving as TAM precursors. Circulating monocytes are recruited in gliomas via several growth factors and chemokines (like CSF-1, CCL2, CCL7, CCL20) [111]. In the TME, they differentiate into TAMs, adopting an M2-like pro-tumorigenic phenotype [117]. Glioma cells can reprogram normal CD14+ monocytes into cells that function as monocytes with immunosuppressive properties. These monocyte-MDSCs (M-MDSCs) are characterized by reduced CD14 but maintained CD11b expression and increased expression of IL-10 and TGF-β [111,117]. Monocytes express a wide range of PRPs including TLRs, NLRs, RLRs and CLRs which in glioma TME enable immunosuppression (through IL-10 and TGF-β cytokine release), fuel inflammation (through the secretion of IL-1β and IL-18) and preserve tumor growth (through VEGF, MMPs, and IL-1β expression) [113,114,115,117,118,119,120].

### 3.4. Natural Killer Cells

NK cells are critical members of innate immunity, expressing several innate immune receptors (TLRs, NLRs, RLRs and CLRs). Their activity is often suppressed in glioma TME, leading to impaired tumor surveillance [121]. A downregulation of NKG2D ligands (such as MICA/B, ULBPs) along with secretion of immunosuppressive factors (like TGF-β, PGE2) from glioma cells has been shown to block the cytotoxic function of NK cells [122].

In addition, TLR-induced immunosuppressive cytokines (such as TGF-β, IL-10) released from glioma-associated myeloid cells have been shown to impair NK function [113,114]. Moreover, the activation of NLRP3 inflammasome activation results in IL-1β secretion enhancing glioma progression but also recruiting NK cells which can be further activated by type I interferons through RIG-I/MDA5 signaling [123], indicating that innate immune receptors may induce both activation as well as suppression of NK cells in glioma TME.

### 3.5. Neutrophils

Neutrophils infiltrate gliomas in large numbers, exerting both anti-tumor (N1) as well as pro-tumor (N2) phenotypes. Gliomas, however, mainly polarize neutrophils towards the N2 phenotype promoting angiogenesis by secreting growth factors such as VEGF, suppressing T cells and inducing remodeling of extracellular matrix [124]. Through TLR2/4 signaling, neutrophils release cytokines such as IL-8, CXCL1, and CXCL2, which induce further recruitment of neutrophils into the TME. TLR ligands that are present in glioma cells induce neutrophils reprogramming to a pro-tumorigenic state [125]. In addition, NLRP3 activation in neutrophils induces IL-1β and IL-18 secretion, promotes chronic inflammation and enhances tumor growth. Furthermore, neutrophil infiltration contributes to tumor progression via immunosuppression and can give rise to polymorphonuclear MDSCs (PMN-MDSCs) [113,114,126]. They differ from conventional neutrophils as characterized by their low-density and lipoprotein receptor-1 (LOX-1) expression. These findings suggest that neutrophils and PMN-MDSCs play a role in both supporting glioma growth and TME suppression [113,114,125,126].

### 3.6. Cytokine Networks

Cytokines are essential in facilitating immune responses within the glioma TME. Acting across intratumoral, perivascular and CSF compartments, they regulate immune cell recruitment, differentiation, effector suppression and angiogenesis [127,128]. TAM-associated cytokines include pro-inflammatory mediators such as TNF-α, IL-1B, IL-6 and IL-12 and anti-inflammatory mediators including IL-10 and TGF-β [34,104,109,110]. These factors impact the activity of T cells, macrophages and tumor progression.

A major immunosuppressive cytokine in the glioma TME is IL-10. Its suppressive effects on T cell activity and enhanced M2-like macrophage polarization leads to tumor growth and immune evasion through dampening of antitumor immune responses [127]. IL-1β promotes inflammation and facilitates tumor progression by promoting angiogenesis and attracting immunosuppressive cells. Additionally, it establishes a pro-inflammatory environment that aids tumor survival [128]. IL-6 is a pro inflammatory cytokine that enhances the expansion of MDSCs. Heightened levels of IL-6 in the glioma TME have also been linked with enhanced tumor growth and apoptotic resistance. All three collectively contribute to a pro-tumorigenic glioma TME, despite IL-10 being anti-inflammatory and IL-1β and IL-6 being pro-inflammatory.

The interplay between anti-tumorigenic and pro-tumorigenic cytokines has significant implications towards immune regulation in the glioma TME. Anti-tumorigenic cytokines coexist in abundance in glioma’s TME. A notable example is type I IFNs, which enhances DC maturation and cross-priming of CD8+ T cells, upregulates MHC-I and can sensitize gliomas to a checkpoint blockade [128]. Another example is IL-12, which skews responses toward a Th1 phenotype and induces IFN-γ, activating NK cells and CTLs and reducing tumor growth. Its clinical use is limited by toxicity, however, highlighting the need for targeted delivery [127].

The dualistic nature of cytokine signaling in gliomas highlights areas for further research, both in regard to tumor growth as well as to its reduction. Targeting these cytokine networks can offer therapeutic opportunities to shift the balance towards an antitumor immune response, allowing for better treatment approaches [129].

## 4. Challenges and Therapeutic Strategies

The cancer immunity (CI) cycle refers to the steps involved in antigen release and DC presentation required to the activation of T cells to the TME [86,87,88]. Disruptions to the CI have been found in GBMs, most notably in the steps of antigen presentation and release where poor tumor antigen availability, DC dysfunction and MHC downregulation impair T cell activation. CSF1R-dependent TAM-mediated suppression and corticosteroids further suppress immune activation within the TME as well as the draining of lymph nodes [86,88]. Research studies demonstrate that meningeal lymphatic vessels (MLVs) may serve a vital function in the presentation of antigens and downstream immune activation, suggesting its consideration in future treatment approaches [86,87].

One of the paramount challenges in targeting innate immune receptors lies in their dual roles within the glioma TME. Depending on the cellular context, the activation of these receptors can display both supportive and inhibitory effects on the tumor, complicating the process to develop therapeutic targeting. For instance, while certain innate immune pathways can stimulate anti-tumor immunity, their activation may also promote inflammation and immune evasion, as observed in gliomas [130]. This manipulation of both innate and adaptive immune systems involves a coordinated release of cytokines, chemokines, and other molecular signals, which suppress immune cell activity and ultimately promote tumor survival and growth [131].

The STING pathway is often inhibited by gliomas through the reduced expression of key components such as cGAS and STING, leading to impaired detection of tumor-derived DNA and lowered production of type I IFN, found to be essential in triggering tumor suppressive responses [132]. Additionally, tumor-derived factors, including immunosuppressive cytokines like TGF-β, further suppress STING signaling, creating significant obstacles to effective immune activation [132].

STING activation triggers type I IFN synthesis and is key in promoting anti-tumor immunity by enhancing DC maturation and activating T cells. However, if STING activation is prolonged or excessive, it has the potential to cause chronic inflammation. The inflammatory environment then mobilizes cells to suppress immunity, such as MDSCs and Tregs, ultimately facilitating tumor progression. Given that glioma-associated macrophages exert both tumor-killing as well as tumor-promoting roles, this further highlights the dual roles within the TME [46].

Gliomas further complicate this dynamic relationship by employing significant immune evasion strategies. These tumors downregulate innate immune receptor expression or their signaling pathways to avoid detection and activation of immune responses. Additionally, gliomas secrete immunosuppressive agents, notably TGF-β and IL-10, to create a suppressive TME, thereby dampening the effects of innate immune receptor activation [130,132]. Due to the complexities associated with the dual functions of receptors and cytokines, addressing the challenges posed by immune evasion in gliomas demands further innovative strategies.

While most innate receptors in gliomas drive tumor growth and/or immune evasion, some receptors, such as RIG-I and MDA5 have shown anti-tumor activity via type I IFNs and apoptosis. This suggests that future therapies should explore a combination of upregulating certain anti-tumor receptors, while simultaneously blocking pro-tumor receptors [55,56,96,97].

Another challenge faced is the permeability of the blood-brain barrier (BBB). Due to the presence of continuous tight and adherens junctions, particles may reach the brain tissue by moving across the endothelial cell luminal and the abluminal plasma membranes, obstructing the dispersal of many oncologic drugs and monoclonal antibodies. On the other hand, physically disrupted regions of the BBB can be used to detect dense tumor regions, presenting the primary targets for surgical resection upon detection by gadolinium-contrast MRI [33].

An additional therapeutic strategy being explored is the use of a nanoparticle (NP) drug delivery system (NDDS) due to their distinct features, such as biodegradability with minimal side effects. Following the disruption of the BBB, a blood-tumor barrier (BTB) which exhibits higher permeability than the BBB will emerge, allowing NP penetration and accumulation at the tumor site. Furthermore, it extends the drug efficacy by blocking the lymphatic reflux in the tumor region, enabling it to remain in the tumor tissue for a prolonged period of time. Its effectiveness is limited by several factors, the most significant being that it requires improvement in the field of NP delivery and drug therapy [42].

Regardless of their limitations, progression by surface modification and combination with ligands and peptides offers potential in the treatment of GBM in patients, especially with the ongoing advances of nanotechnology and extensive research [42].

The rise of immunotherapy to combat cancer cells has shown significant promise in the treatment of gliomas. The main immunotherapies being investigated for treatment include immune checkpoint inhibitors (ICIs) which play a complex function in aiming to enhance T-cell mediated anti-tumor response that is subsequently suppressed by the TME. Chimeric Antigen Receptor T-cell (CAR-T) therapy employs the modification of T cells to target specific markers on tumor cells. Treatment with oncolytic viruses have been shown to selectively target tumor cells and was recently approved in Japan [133].

Lastly, there is the use of vaccines to deliver antigens. Immunotherapy is a promising approach in glioma treatment; as researchers continue to tackle limitations, improvements in immunotherapy may promise more favorable outcomes for patients in the near future.

## 5. Conclusions

Innate immune receptors play an instrumental role in the development of gliomas, the progression of tumors, and immune regulation. Innate immune receptors contribute to glioma proliferation, invasion and immune regulation in context-dependent ways. As discussed in Section 2 and Section 3, receptors such as TLR2, TLR4, TLR7, TLR9 and NLRP3 generally promote tumor progression and immune suppression. In contrast, RIG-I, MDA5, TLR3 and STING can include antitumor mechanisms via type I IFNs and apoptosis. Scavenger receptors as well as CLRs can further promote immune evasion by tolerizing antigen presentation and can alter the activity of TAMs. Their dual nature makes targeting these receptors both a challenge as well as an area of potential development. Gliomas enhance this complexity by actively suppressing receptors through strategies including reducing their signaling, driving a complex cytokine milieu and mobilizing MDSCs and Tregs to weaken the immune response [113,114,125,130].

Each receptor has a unique contribution to shaping the glioma immune environment. TLRs activate inflammatory responses but can also promote tumor-supportive cytokines like IL-6 [125]. Through inflammasomes like NLRP3, NLRs can create an inflammatory environment that suppresses the region’s immune activity [114]. Pathways such as RLRs and STING show anti-tumor potential through type I IFN production along with recruitment of immune cells; however, these pathways are often suppressed by gliomas to varying degrees [126,130]. Scavenger receptors and CLRs also play a role in helping tumors evade immune detection by clearing tumor antigens and dampening immune activation [75,83]. The complex and often conflicting ways that these receptors and immunocytes engage, depending on the environment, TAMs, MDSCs, as well as cytokine networks, further emphasize their significance in the progression of gliomas [104,114].

Despite these challenges, innate immune receptors remain valuable targets for therapeutics. Preclinical studies involving STING agonists, TLR modulators, and NLRP3 inhibitors are being studied in regard to how they alter the TME in gliomas [130]. When used in conjunction with therapies that block inhibitory immune pathways and traditional treatments, namely chemotherapy and radiation, these emerging therapies have shown potential in strengthening immune responses [132]. Personalizing treatments based on tumor-specific receptor profiles offers even more promise, as this is able to address the unique characteristics of the specific tumor [130]. Improvements in drug delivery methods, such as nanoparticles, are also ameliorating the precision and effectiveness of these novel treatment methods [75].

While there are promising developments in the therapeutic realm, challenges remain and will continue to evolve. Depending on cellular context, innate immune receptors can facilitate pro-tumor as well as anti-tumor responses. This makes it difficult to design treatments that boost immunity without unintentionally promoting tumor growth. Gliomas’ diverse nature and highly suppressive environment add further complications. Overcoming these issues will require deeper research of these receptors’ function and their modulation for therapeutic benefit [128,130].

Looking ahead, future research should focus on developing new receptor modulators and testing them in combination with other traditional therapies. Clinical trials that combine receptor-targeted therapies with ICIs and standard treatments have potential for turning preclinical findings into real-world solutions [130]. Additionally, improving methods to deliver drugs effectively to the brain will be a key step in making these therapies more successful [75].

Targeting innate immune receptors presents both a challenge and an opportunity in gliomas treatment. By finding ways to utilize their anti-tumor potential while overcoming the barriers presented by the TME, researchers can strive in a direction that improves outcomes for glioma patients. The combination of advances in immunotherapy, personalized medicine, and novel delivery systems offers hope for combating this devastating disease [130,132].

## Figures and Tables

**Table 2:** Overview of innate immune receptors involved in glioma development.

Receptor Type	Ligands/Triggers	Key Signalling Pathway(s)	Pro-Tumor Effects	Anti-Tumor Effects	Immune Evasion Mechanisms	References
TLRs	PAMPs, DAMPs	NF-κB, MAPK	IL-6, TNF-α	Apoptosis (via TLR3), IFN production	Downregulation, altered TLR expression	[14,16,17,18,29,90,91]
NLRs	Intracellular DAMPs	Inflammasomes (NLRP3)	IL-1β secretion, immune suppression	Immune recruitment (Context dependent)	Chronic inflammation, TME shaping	[48,49,52,92,93,94]
RLRs	Viral RNA	IRF3, type I IFNs	Some evasion mechanisms	Induces apoptosis, IFN signalling	Tumor-mediated signalling suppression	[54,55,56,95,96,97]
STING	Cytosolic DNA	TBK1, IRF3	Chronic activation → MDSCs, Treg	Type I IFNs, T cell activation	STING/cGAS downregulation	[58,59,62,66,70,72,98,99]
Scavenger Receptors	AGEs, AOPPs, lipids	Numerous	Antigen clearance, immune suppression	-	Tolerogenic signalling, TME shaping	[75,78]
CLRs	Pathogen Carbohydrates	SYK, RAF1	Tolerogenic DCs, IL-10 production	Context-specific	Exploitation by tumors to dampen immunity	[83,84,85,100,101,102]

## Data Availability

Not applicable.
